# The importance of size, location, and vegetation composition of perennial fallows for farmland birds

**DOI:** 10.1002/ece3.4420

**Published:** 2018-08-24

**Authors:** Kim S. Meichtry‐Stier, Jérôme Duplain, Michael Lanz, Bernard Lugrin, Simon Birrer

**Affiliations:** ^1^ Swiss Ornithological Institute Sempach Switzerland; ^2^ Chemin de Champ‐Manon 33 1233 Bernex Switzerland

**Keywords:** agri‐environment scheme, arable, biodiversity, field margin, set aside, Switzerland, trends, wildflower area

## Abstract

Across Europe, patches of un‐cropped land (field margins, fallows, etc.) have been established and managed as part of agri‐environment schemes (AES) to counteract the decrease in farmland biodiversity. Various studies demonstrate a positive impact of such un‐cropped land on different taxa. However, there is potential to further improve the efficiency of fallow options for farmland birds. In a long‐term monitoring, 12 breeding farmland bird species and sizes of perennial fallows were recorded from 1992 to 2015 in a 6.1 km^2^ area in Switzerland. Furthermore, habitat composition and fallow characteristics were mapped in 2012. We calculated population trends, analyzed habitat associations and revealed the impact of fallow habitat characteristics on territory density. The proportion of fallows in the study site increased from 1.4% (1992) to 8.5% (2012). Population trends of six of 12 censused species increased significantly over the same time, four species showed no trend and trends of two species decreased. Seven species were analyzed in more detail, for five of them fallows were overrepresented around their territory center points compared to arable fields and grassland. The overall territory density of these five species was higher in small fallows which were not placed next to a wood and which held bramble *rubus* spp., shrubs and the tall‐growing forb goldenrod (*Solidago canadensis* and *S. gigantea*). Our study confirms that perennial fallows are a highly suitable option to support different farmland birds in arable landscapes. Yet, we recommend optimizing fallows through careful site selection and management, such that they are not established on shady locations and are structurally diverse by allowing brambles, shrubs, and tall‐growing forbs to occur. We suggest adapting the Swiss AES in this regard. Biodiversity‐related advisory services available for farmers could increase the probability that fallow options are implemented and managed properly for targeted species.

## 1 INTRODUCTION

Populations of farmland birds have been severely decreasing in Europe over the last decades (PECBMS 2016), mostly due to the agricultural intensification, which led to habitat degradation and loss (Benton, Bryant, Cole, & Crick, 2002; Donald, Sanderson, Burfield, & van Bommel, 2006; Shrubb, 2003). Hedgerows were cleared, rural, or overgrown uncultivated areas disappeared and formerly extensively used crop margins impoverished ecologically through the applications of herbicides and other pesticides. To halt or reverse the decline of farmland biodiversity, agri‐environment schemes (AES) were developed in most European countries. Several of these AES provide an option of un‐cropped land (land that otherwise could be cultivated) that is managed specifically to provide benefits for wildlife. In the existing literature, the most frequently used terms are “field margin” and “fallow,” covering a whole range of options such as beetle banks, cultivated/arable margins, wildflower strips, grassy boundaries, sown grass strips, ditch banks, rotational fallows, conservation headlands, fauna margins (Cordeau, Petit, Reboud, & Chauvel, 2012; Emerson & Gillmor, 1999; Haenke, Scheid, Schaefer, Tscharntke, & Thies, 2009; Maddock, 2008; Marja & Herzon, 2012; Marshall & Moonen, 2002; Noordijk, Musters, van Dijk, & de Snoo, 2010; Vickery, Feber, & Fuller, 2009; Zollinger, Birrer, Zbinden, & Korner‐Nievergelt, 2013). Field margins are usually a linear element at the edge of an arable field. The term “fallow” comprises both, strips and larger parts of a field. It can be managed similarly to field margins, for example, using the same seed mixes, but often stays only for one vegetation period. In Switzerland, six AES options on arable land exist, namely wildflower area/strip, rotational fallow, conservation headland, improved field margin, and flower strips (Caillet‐Bois, Weiss, Benz, & Stäheli, 2018) as well as naturally vegetated field margins as part of a local AES. In this study, we concentrate on the three AES types sown wildflower area, rotational fallow, and naturally vegetated field margin and named them “fallows.” In contrast to fallow options in other countries, in our study site the fallows were perennial (lasting for more than one vegetation period). Being quite divers in spatial configuration, they were established with the aim to create fallow‐like habitat by increasing structural and botanical richness in arable landscapes, thereby, for example, provisioning breeding habitat or food sources for birds.

**Figure 1:**
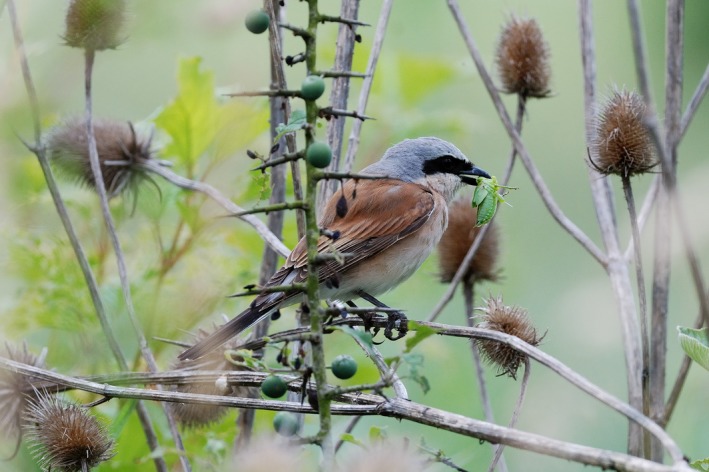
Red‐backed Shrike on a Wild Teasel, photo: Markus Jenny

Various studies report a positive influence of field margins and fallows on birds (Burgess et al., 2015; Henderson et al., 2012; Kuiper, 2015a,b; Meichtry‐Stier, Jenny, Zellweger‐Fischer, & Birrer, 2014; Tryjanowski, 1999) and other species (Haaland, Russell, & Bersier, 2011; Meichtry‐Stier et al., 2014; Van Buskirk & Willi, 2004; and references therein). Despite these benefits, farmland bird indices are decreasing in the EU (PECBMS 2016) and Switzerland (see “Swiss Bird Index—Priority Species Agriculture” in Sattler, Knaus, Schmid, & Strebel, 2016). Possible reasons for this are low floristic and structural diversity (ecological quality) of implemented options and the low uptake of the most appropriate options by farmers (Birrer, Spiess, Herzog, Kohli, & Lugrin, 2007; Breeze, Bailey, Balcombe, & Potts, 2014; Hardman et al., 2015). A few studies described specifically what these qualities are for biodiversity and birds in particular. A management that “creates a structurally and floristically diverse sward” thereby providing resources such as insects and seeds as well as the “proximity to a good quality hedgerow” of field margins and fallows were shown to be beneficial for birds (Tscharntke, Batáry, & Dormann, 2011; Vickery et al., 2009). Different results are found about the optimal age and size of un‐cropped land for birds, reflecting the diverse local conditions and landscape history (Flade, Plachter, Schmidt, & Werner, 2006; Henderson et al., 2012; Holland, Storkey, Lutman, Henderson, & Orson, 2013; Tscharntke et al., 2011; Zollinger et al., 2013). In terms of structural and floristic diversity, implemented options often look too similar to the crops around instead of creating an ecological contrast. Fallows in Switzerland are designed to increase the floristic and structural diversity (Figure 1) and thus stand out from the neighboring uniform farmland, providing resources (e.g., food, nesting opportunities, or song posts) that are limited in the surroundings for birds (Batáry, Dicks, Kleijn, & Sutherland, 2015; Hammers, Müskens, van Kats, Teunissen, & Kleijn, 2015). Yet, the influences of the vegetation composition of un‐cropped land on birds have been much less investigated (but see Marja & Herzon, 2012). Un‐cropped land undergoes succession and especially in older ones, small shrubs, bramble or goldenrod may appear, or mainly grassy vegetation develops.

In Switzerland, fallows are often suboptimally placed on wet, steep, or shady areas (Herzog et al., 2005). There, botanical diversity is impaired because many species of the seed mix do not grow and are outcompeted by a few dominating species. We assume that many fallows could be optimized in location, size, and vegetation composition. To improve fallows for conservation management, more knowledge is needed about the preferred characteristics of such habitats for breeding farmland birds.

The study is embedded in a long‐term monitoring of priority farmland birds (birds of conservation concern according to the Swiss legislation) in an ecologically improved landscape. In this project region, perennial fallows were established, providing the possibility to study the relationship between farmland birds and characteristics of the fallows. Our questions were threefold: (a) Do abundances of priority bird species increase over the long‐term study period? We expect the trends to be more positive than European or Swiss bird indices, because of the comparably high percentage of fallows in the study region. (b) Do territories of priority farmland bird species contain more fallow area than expected according to the available habitat in the study site? Studies from other regions and countries revealed a preference for fallow habitat. (c) Which characteristics of fallows are related to territory density of priority farmland birds and is vegetation composition of the fallows related to territory density? Personal observations revealed that small shrubs, brambles, and goldenrod increase the attractiveness of fallows whereas a high amount of grass is less favored by priority bird species of open farmland.

## 2 METHODS

### 2.1 Study site

We conducted our study in the very southwest of the Swiss lowland, the Champagne genevoise (420–450 m a.s.l., 46°09′11″N, 6°01′25″E, canton Geneva), which has a warm and dry climate (mean temperature is 11.2°C, mean yearly precipitation is around 700 mm). The study site (6.1 km^2^) is dominated by intensively cultivated farmland (mainly winter cereals) and some large gravel pits (covering between 6.1% and 13.2% of the study area in all years) and holds only a few small settlements and woods (Table 1). The depleted gravel pits were filled up and then used again as farmland. Some hedgerows and small coppices structure the wider countryside, the hedges being quite diverse in density and height but mostly lower than 3 m. The landscape composition within the study site is simple (Tscharntke et al., 2011) with less than 20% semi‐natural habitats (extensively used meadows and pastures, ponds, hedgerows, woods, ruderal areas). Mean field size is small (1.0 ha) compared to other arable landscapes of Western Europe. Nonetheless, the area is intensively farmed with fields under annual crops and infrequent hedgerows. The only common semi‐natural element is narrow grassy margins (approx. 1 m wide) along the roads.

**Table 1:** Description and proportion of the habitat categories in the study site in 2012, as used in the compositional analysis

Category	Description	Proportion of study area (%)
Fallow	Naturally vegetated perennial un‐cropped strips on arable farmland from a local AES, sown wildflower areas or sown rotational fallow from the Swiss AES (either as strip or area); fertilization and treatment with insecticides are not allowed; large‐scale chemical or mechanical weed control is not allowed; cut max. once a year on half of the area	8.5
Hedgerow	Hedgerows within farmland	0.8
Arable	Farmland used for conventional arable production, mostly winter wheat (21.5% of the study area), winter rapeseed (9.2%), winter barley (7.4%), and sunflowers (5.7%). Treatments with fertilizers and agrochemicals allowed.	53.0
Wood	Small woods and coppices	1.7
Gravel	Gravel‐pits and small ruderal areas not on farmland	13.0
Grassland	Meadows and pastures; semi‐natural fields (mown once a year and not fertilized) as well as intensively used grassland (mown several times a year and fertilized), some grazed with horses.	12.4
Others	All other areas, for example, roads, settlements, vineyards, pomiculture, ponds	10.6

### 2.2 Fallows

In this study, fallows were either sown wildflower areas/strips, rotational fallows, or naturally vegetated field margins, showing different characteristics of structural and floristical diversity. Wildflower areas/strips and rotational fallows are options of the Swiss AES (Caillet‐Bois et al., 2018), naturally vegetated field margins are part of a local AES. They are designed to halt the overall biodiversity loss in farmed landscapes without a special focus on birds or even single species. Since 1992, perennial fallows have been implemented by farmers in the study site, mostly according to instructions regarding site selection and management by one of the co‐authors (BL). In 2012, there were 82 fallows next to 189 arable fields (Table 1). They were either naturally vegetated (left fallow after harrowing) or were sown with species‐rich wildflower seed mixes (minimum 17 plant species). In some of them, shrubs and bramble were cleared, in others bramble, shrubs (mainly common dogwood *Cornus sanguinea*, small willows *Salix* spp. and small poplars *Populus* spp.) and goldenrod (mainly *Solidago gigantea*, but also *S. canadensis*) occurred in different amounts therein.

Every few years, they were partially mown to reduce the expansion of goldenrod, bramble, and shrubs. Their spatial configuration was quite diverse, most of them being linear strips of 10–25 m width; others were of rectangular (whole parcels) or triangular shape. Size ranged from 0.06 to 3.3 ha (median 0.36 ha). Their age ranged from zero to 22 years (median 13 years). The use of herbicides and pesticides was not allowed. All these fallows stand out from the surrounding arable fields by their high botanical and structural richness. They usually bordered directly on crop fields or meadows with no physical boundary in‐between and only 15 were adjacent to hedgerows. Some of them adjoined roads, gravel‐pits, ruderal areas, or small woods. The proportion of fallows relative to the area of the study site increased significantly from 1.4% in 1992 to 8.5% in 2012 (linear regression, estimate = 0.35, *df* = 14, *t* = 22.9, *p* < 0.001, *R* ^2^ = 0.97).

### 2.3 Data collection

In the long‐term monitoring, we censused territory numbers of 12 breeding farmland bird species of conservation concern (Common Whitethroat *Sylvia communis*, European Stonechat *Saxicola torquata*, Melodious Warbler *Hippolais polyglotta*, Yellowhammer *Emberiza citronella*, Red‐backed Shrike *Lanius collurio*, Corn Bunting *Miliaria calandra*, Cirl Bunting *Emberiza cirlus*, Common Quail *Coturnix coturnix*, Western Yellow Wagtail *Motacilla flava*, Ortolan Bunting *Emberiza hortulana*, Northern Lapwing *Vanellus vanellus*, Whinchat *Saxicola rubetra*). Eight of the species are listed as “Species of European Conservation Concern” (SPEC). All species are listed in the environmental objectives of the agricultural sector in Switzerland (EOA species, BAFU & BLW 2008), except the Melodious Warbler. This species is of local conservation concern as the project region holds a major part of the Swiss population. Other EOA species were not censused yearly because a yearly census would have been too time‐consuming (skylark), or the species were not typical for open arable farmland in Switzerland (nightingale, turtle dove). Bird census took place from 1992 to 2015 except for 2006. Yellowhammer and Cirl Bunting were mapped from 1994 and 1995 on. In addition, Skylark was mapped in 2012 to include this typical and locally abundant field nesting species in the compositional analysis (see further down). Each year, the study site was visited six times from 5 to 9 a.m. between April and July. Monitoring was only performed in suitable weather conditions (minimal wind, no rain). The same observer (BL) was sampling in all years, except 2010, sometimes supported by other ornithologists. Monitoring was conducted by walking along paths which allowed audio‐visual observation of the entire study site. All observations of each bird species were recorded on maps following the breeding bird census method (Bibby, Burgess, Hill, & Mustoe, 2000; Schmid, Zbinden, & Keller, 2004). To assess breeding territories, species had to be observed during their breeding period in a suitable habitat (Schmid, Luder, Naef‐Daenzer, Graf, & Zbinden, 1998), at least twice during different survey rounds or displaying territorial behavior (e.g., song, conflicts indicating territory defense, transport of nest material). Territory center points were specified as geometric center point of the observations of each territory with ArcGIS.

Each year from 1992 to 2015, size of fallows was recorded. For the compositional analysis and the habitat model (see further down), a habitat mapping was conducted in 2012. Land‐use types of the whole study site were mapped between June 5th and August 10th, assigning each type to one of the habitat categories in Table 1. Then, each fallow was surveyed in‐depth, determining structural variables and vegetation composition (Table 2). Distance to the nearest wood was defined using the compiled map of land‐use types and orthophotos (SWISSIMAGE © 2016 swisstopo DV 043734). The proportions of bramble, goldenrod, and grass per fallow were visually estimated during field visits by the same fieldworker, traversing through every single fallow. Strips were crossed once, and bigger fallows were traversed along two or three lines. The amount of shrubs (woody plants of relatively low height, lacking a single trunk; not including brambles) was defined the same way but due to the strongly skewed distribution of this variable, the values were grouped in three categories (0%, 1%–10%, >10%).

**Table 2:** Description of the explanatory variables (structural variables and vegetation composition of fallows) used in the habitat model (GLM)

Variable	Description	Range, median
Size	Area of the fallow, measured in ha	0.06–3.3, 0.36
Age	Age of the fallow (in years)	0–22, 13
Distance to wood	Distance from the center of the fallow to the nearest wood (in meter)	10–400, 130
Shrubs	Amount of shrubs (e.g., dogwood, willow, poplar) in the fallow	0: 0%, 34 fallows 1: 1%–10%, 32 fallows 2: >10%, 16 fallows
Bramble	Proportion of the fallow overgrown with bramble	0%–85%, 2%
Goldenrod	Proportion of the fallow overgrown with goldenrod	0%–90%, 13%
Grass	Proportion of the fallow overgrown with grass	0%–95%, 13%

### 2.4 Statistics

All analyses were performed in R 3.3.1 (R Development Core Team 2015).

For each species, the population trend was calculated with a generalized linear model (GLM, package “arm”) using a Poisson distribution. Territory numbers (response variable) were tested against year and the second and third polynomial of year to account for a possible change in the population trend within the study period. The polynomials of year were removed from the full model if the BIC of the model with the polynomial(s) was less than 2 units lower than the BIC of the reduced model. Overdispersion appeared in the models of Corn Bunting and Common Quail; thus, for these species, we calculated a generalized linear mixed model (GLMM) instead, including observation number (unique number of each row in the data set) as a random factor. Model fit was assessed graphically by plotting the residuals against the fitted values and by plotting the residuals against each explanatory variable.

We analyzed the association of birds with the different habitat categories in 2012 (Table 1) with a compositional analysis (Aebischer, Robertson, & Kenward, 1993; Calenge, 2011; package “adehabitatHS”). This was performed for the seven species with more than 20 territories (based on the recommended sample sizes in Aebischer et al., 1993): Common Whitethroat, European Stonechat, Melodious Warbler, Yellowhammer, Red‐backed Shrike, Corn Bunting, and Skylark. Around each territory center point a buffer (radius 50 m) was laid. Subsequently, we term these buffer areas pseudoterritories. We chose 50 m for two reasons: (a) this way each pseudoterritory covers an area of 0.8 ha, which corresponds to the known territory sizes of the three studied species Whitethroat, Stonechat, and Yellowhammer (Bauer, Bezzel, & Fiedler, 2005); (b) For Red‐backed shrike, Corn bunting, and Skylark the pseudoterritories of 0.8 ha are smaller than their known territory sizes. However, a larger buffer radius would result in many overlapping pseudoterritories. Only pseudoterritories lying completely within the study site were used for the compositional analysis. Then, the proportions of each habitat category within each pseudoterritory and within the whole study site (= available habitat) were calculated. Zero values in the pseudoterritories were replaced by 0.001. Randomization tests (with 500 replicates) were used for both the significance of habitat association (using Wilks lambda) and of the habitat ranking. Habitat categories were ranked independently of availability, indicating whether a habitat category was significantly more or less represented in the pseudoterritory than the other categories.

To find out which characteristics of fallows were related to territory density, we calculated a habitat model using the data on vegetation composition of fallows also collected in 2012. Because the Swiss fallows are designed to increase the diversity and total abundance of farmland species rather than promoting single species, we pooled territories of the five species which showed a clear association with fallows in the compositional analysis (Whitethroat, Stonechat, Melodious Warbler, Yellowhammer and Red‐backed Shrike). We analyzed the relationship of territory density within each fallow (*n* = 82) to structural variables as well as vegetation composition of each fallow. This was performed with a generalized linear model (GLM, package “arm”) using a Poisson distribution with log link function and the number of territory center points per fallow as dependent variable. Territory center points that did not fall into a fallow were counted as well if they were laying 10 m at most outside a fallow. Explanatory variables were size, age, distance to wood, and amount of shrubs, proportions of bramble, goldenrod, and grass. Because fallows were of different sizes, we included the logarithm of size (in ha) as offset in the model. Thus, we modeled territory densities (number of territories per ha) rather than absolute numbers of territories. The proportions of bramble, goldenrod, and grass were arcsine‐square root‐transformed and distance to wood was square root‐transformed. All explanatory variables were standardized with mean zero and *SD* = 1. No collinearity was found between the explanatory variables, as variance inflation factors were all <3 (Zuur, Ieno, Walker, Saveliev, & Smith, 2009). Quadratic polynomials of size, age, distance to wood, bramble, and goldenrod were included in the model because we expected an optimum for these variables, or for size even another nonlinear relationship. From this full model, the polynomial terms of age and bramble were removed because their 95% credible interval (CrI; calculated by Bayesian statistics, see procedure in Zollinger et al., 2013) did not include zero. The interpretation of lower‐order polynomials is difficult when higher‐order polynomials are present. Thus, the elimination of nonsignificant higher‐order polynomials makes the lower‐order polynomials interpretable. Linear effects of the variables were not selected out of the model as we had an a priori interest in these variables. Moreover, eliminating nonsignificant effects may lead to biased parameter estimates (Whittingham, Stephens, Bradbury, & Freckleton, 2006). Variables were considered significant if their 95% CrI did not include zero. No spatial autocorrelation was found in the final model (checked by variogramm and bubble plot). We graphically assessed if model assumptions were met, as described above.

## 3 RESULTS

### 3.1 Population trends

**Figure 2:**
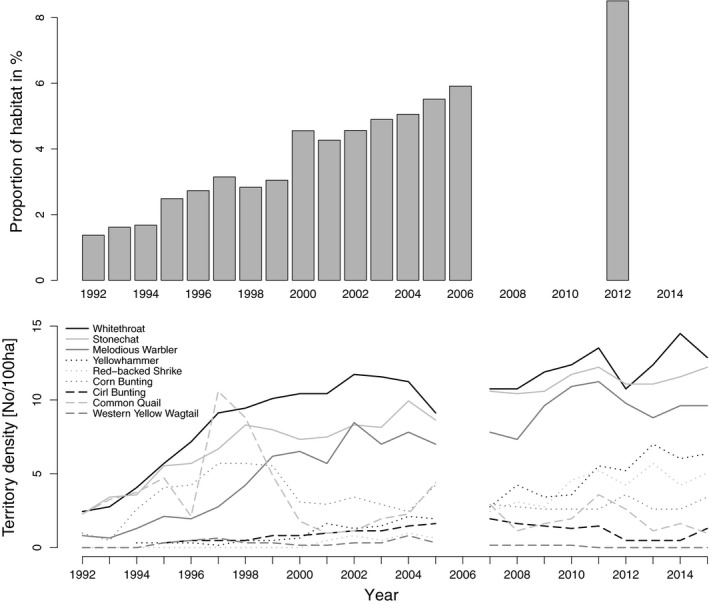
Population trends of nine farmland bird species (lines) from 1992 to 2015 and change in total fallow area (bars) as a proportion of the study region (6.1 km^2^). Fallows were mapped in the years 1992 to 2006 and 2012. For clarity reasons Ortolan, Lapwing and Whinchat are not shown, as their territory numbers were zero almost every year and lines would strongly overlap

Territory numbers of six (Whitethroat, Stonechat, Melodious Warbler, Yellowhammer, Red‐backed Shrike and Cirl Bunting) of the 12 yearly censused species in the study site increased significantly during our study (Table 3 and Figure 2). Two species (Ortolan and Whinchat) showed no significant trend, yet they were both nonregular breeders in the study site and occurred only in a few years and in very low numbers. The Corn Bunting and the Western Yellow Wagtail showed (the latter on a low level) an increase for a few years followed by a decrease. Only two species (Common Quail and Northern Lapwing) decreased significantly over the years. However, Quail territory numbers were strongly fluctuating throughout the study and Lapwing numbers had been on a very low level from the beginning of the census.

**Table 3:** Population trends of the monitored bird species in the study site between 1992 and 2015

Species	Estimate of year	Estimate of year^2^	Estimate of year^3^	No. of territories
Common Whitethroat *Sylvia communis*	0.03	−0.25***	0.19***	15–79
European Stonechat *Saxicola torquata*	0.37***	−0.15***	—	14–75
Melodious Warbler *Hippolais polyglotta*	0.43***	−0.43***	0.16**	5–59
Yellowhammer *Emberiza citrinella*	1.28***	−0.27**	—	2–39
Red‐backed Shrike *Lanius collurio*	2.88***	−1.06***	—	0–31
Corn Bunting *Miliaria calandra*	−0.63***	−0.21**	0.38***	6–21
Cirl Bunting *Emberiza cirlus*	0.53***	−0.62***	—	2–8
Common Quail *Coturnix coturnix*	−0.32**	—	—	14–6
Western Yellow Wagtail *Motacilla flava*	−0.77	−1.21**	—	0–0
Ortolan *Emberiza hortulana*	−9.80	—	—	2–0
Northern Lapwing *Vanellus vanellus*	−1.09*	—	—	1–0
Whinchat *Saxicola rubetra*	−1.26	—	—	1–0

*Note*

Estimates of the linear models are given, showing the relation of territory numbers with year. The effect of year^2^ and year^3^ is only shown if significant, else it was removed from the model. Asterisks represent *p*‐values: *<0.05, **<0.01, ***<0.001. Number of territories at the beginning of the population censuses in 1992 (and 1994 for Yellowhammer and 1995 for Cirl Bunting resp.) and in 2015 are given.

### 3.2 Compositional analysis

**Figure 3:**
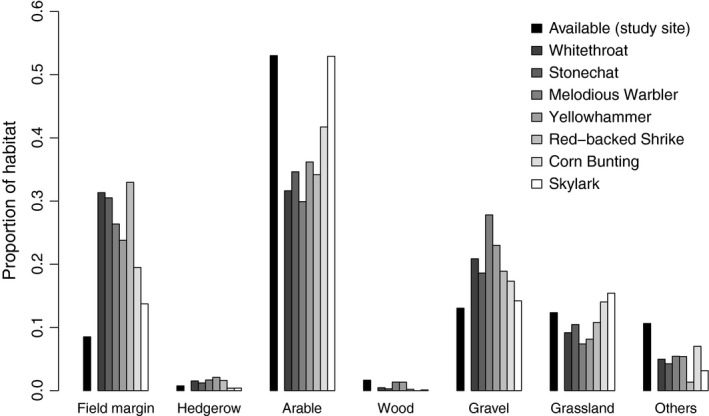
Proportion of habitat in the study site (available) and in the pseudoterritories of the seven farmland bird species analyzed with a compositional analysis for habitat association

The association of the studied species with habitat categories was not random (randomization test, *p*‐values for all species <0.004). For four of the seven analyzed bird species (Whitethroat, Stonechat, Melodious Warbler and Red‐backed Shrike) fallows ranked first before all other habitat categories, that is, fallows were strongly overrepresented in the pseudoterritories (Figure 3, see Supporting Information Appendix S1). The Yellowhammer was associated with fallows and hedgerows. For the Skylark, arable fields, fallows, and hedgerows were ranked highest, whereas the Corn Bunting showed no association with a single habitat category.

### 3.3 Habitat model

**Figure 4:**
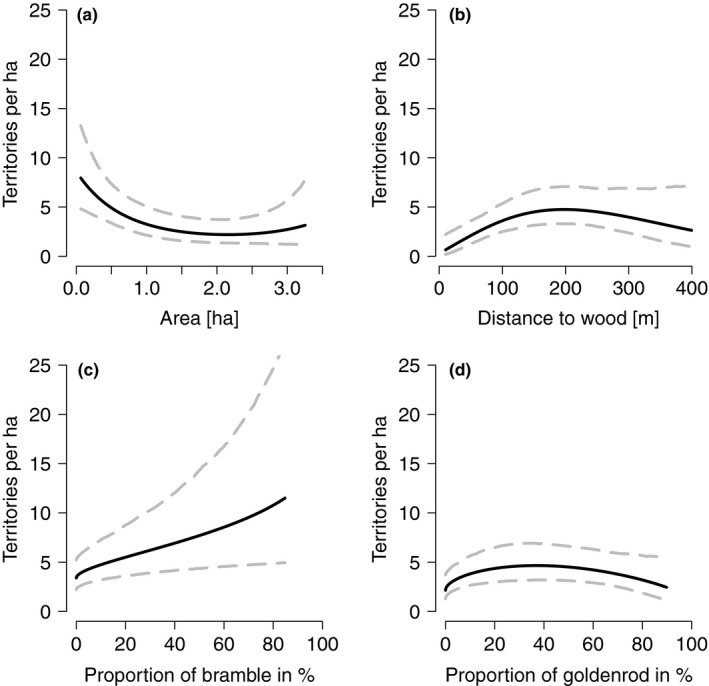
Response curves of habitat variables on territory density of the five species associated with fallows (Whitethroat, Stonechat, Melodious Warbler, Yellowhammer, and Red‐backed Shrike). Effect of (a) area of fallows, (b) distance of fallows to wood, (c) the proportion of bramble in fallows, (d) the proportion of goldenrod in fallows. Values of all other explanatory variables in the models were set to their mean. Dashed lines show the 95% credible intervals

The final model revealed that the density of bird territories was strongly influenced by size, distance to wood, and vegetation composition (proportion of bramble, goldenrod, and shrubs) of the fallows (Table 4). Territory density was negatively correlated with size but positively with the distance to wood and the proportion of bramble (Figure 4a–c). Territory density showed a significant relationship with the proportion of the neophyte goldenrod, with an optimum at approximately 37% goldenrod per fallow (Figure 4d). The positive effect of shrubs on territory density was as strong as the effect of bramble, yet not significant. The least influential (nonsignificant) explanatory variables were age (positive) and the proportion of grasses (negative).

**Table 4:** Estimates and their credible intervals of the final model (GLM) showing the relation between territory density of the five species associated with fallows (Whitethroat, Stonechat, Melodious Warbler, Red‐backed Shrike, and Yellowhammer) and explanatory variables

	Estimate	95% Credible interval		Significance
Intercept	1.46	1.07	1.85	+
Area	−0.57	−0.89	−0.24	+
Area^2^	0.12	0.02	0.22	+
Age	0.10	−0.15	0.34	
Grass	−0.18	−0.39	0.04	
Bramble	0.29	0.07	0.52	+
Shrubs 1%–10%	0.33	−0.09	0.77	
Shrubs >10%	0.32	−0.18	0.84	
Goldenrod	0.28	0.06	0.51	+
Goldenrod^2^	−0.27	−0.50	−0.04	+
Distance to wood	0.30	0.10	0.50	+
Distance to wood^2^	−0.24	−0.46	−0.03	+

*Note*

+: Variables were considered significant if their 95% CrI did not include zero.

## 4 DISCUSSION

Fallows are options of the national and local AES in Switzerland and are designed to promote biodiversity in arable landscapes. In line with former studies, we stress the importance of un‐cropped land like fallows and field margins for breeding farmland birds (Henderson et al., 2012; Meichtry‐Stier et al., 2014; Revaz, Schaub, & Arlettaz, 2008). Most studied species increased in this ecologically improved study site and five of them appear to prefer fallows over arable fields and grassland around their territory center points. These results add to the already known benefits of field margins and fallows for farmland birds. The novelty of the study lies in the suggestions on how the efficiency of fallows can be further improved, with a closer look on vegetation composition. We demonstrate that for the implementation and management of fallows, attention should be paid to the size, location, and the vegetation composition.

Several farmland birds of conservation concern seem to benefit from the ecological improvement of the study region with fallows. The amount of fallows in the study region increased from 1.4% in 1992 up to 8.5% of study site in 2012 (accordingly 11.0% of the utilized agricultural area). In the same period, we measured a large gain in territory numbers of six of 12 bird species. These are species which are usually not increasing in Switzerland or elsewhere in Europe (PECBMS 2016, Sattler et al., 2016), or if so, which have shown a much lower increase than in the study site. The only exception is the Cirl Bunting, whose trend corresponds to the trend in Switzerland and in other European countries. Abundances of Whitethroat, Melodious Warbler, Stonechat, and Yellowhammer increased more than fivefold during 25 years and the Red‐backed Shrike newly occurred. The first three mentioned species reached very high densities (around ten or more breeding pairs per 100 ha) which are records for Switzerland and rare across Western Europe (Bauer et al., 2005; Maumary, Vallotton, & Knaus, 2007). The six species with a positive trend all nest in the herbaceous layer or in shrubs, except the ground breeding Stonechat. In line with former studies showing a positive effect of fallows on birds (Burgess et al., 2015; Henderson et al., 2012; Kuiper, 2015a,b; Meichtry‐Stier et al., 2014; Tryjanowski, 1999), we suppose that their populations benefit from the structurally diverse fallows in the study area which provide dense herbaceous vegetation, bramble, and shrubs. In contrast, territory numbers of Corn Bunting, Whinchat, Western Yellow Wagtail, Ortolan, Common Quail, and Lapwing have not significantly increased, a result which follows the general trends in other countries (PECBMS 2016, Sattler et al., 2016). These species were never numerous in the region and Corn Bunting, Whinchat, and Ortolan decrease even in the nearest optimal habitats (own observations). The other species (Western Yellow Wagtail, Common Quail, and Lapwing) may benefit less from fallows as in our region they have other habitat requirements for breeding such as damp soils, extensively used meadows, or crops with sparse and low vegetation (Maumary et al., 2007). A similar result was found for priority farmland birds in the UK, where mainly species nesting in field boundaries (hedgerows and field margins) benefited from the Higher Level Stewardship management (Bright et al., 2015).

Furthermore, five of the seven studied species (Whitethroat, Stonechat, Melodious Warbler, Yellowhammer, and Red‐backed Shrike) showed a significant association with fallows in the Compositional Analysis. They are typical species of open farmland interspersed with single shrubs (Maumary et al., 2007). Fallows seem to meet their demands. They hold a broad diversity of plant species, many of them flowering and thus providing food for birds by attracting insects (Frank & Reichhart, 2004 and references therein). Thanks to the diverse growth of grass, forbs, and single shrubs, the high structural richness provides shelter and protection against predators for birds and their nests. Also the Skylark, an open field breeder, was associated with fallows, together with arable fields and, surprisingly, hedgerows. This species seems to benefit from fallows, probably using them for foraging while the nest was built in a nearby arable field. Skylarks are known to avoid high structures, but they did not seem to be disturbed by the hedgerows in our study site, most probably because most hedgerows were clearly lower than 3 m. The only species showing no clear association with any habitat category was the Corn Bunting, maybe due to the small sample size. Yet, in 2012 12 of the 22 territory centers lay in fallows and a previous analysis in the region had shown a significant preference of Corn Bunting for fallows between 1992 and 1996 (Jenny et al., 2002).

Our results highlight the important characteristics of fallows for farmland priority bird species and therefore how to optimize this AES option. Size, location (distance to wood), and vegetation composition (amount of bramble and goldenrod, occurrence of shrubs) had the strongest influence on territory density. The effect of age and the amount of grass were less pronounced.

We found a negative effect of fallow size on territory density per fallow. The same relation was found by Zollinger et al. (2013) in western Switzerland. Strips can hold more territories per ha than larger (rectangular) areas because the birds also use habitats around the strip as foraging habitat and only a part of the territory (and the territory center) lies within the fallow strip. Yet, in strips or small areas, the risk of predation may be higher than in large areas due to the increased proportion of edge area (Donald, Evans, Pain, Muirhead, & Buckingham, 1998; Suvorov, Svobodova, & Albrecht, 2014). Predators prefer to roam along edges and linear structures like field margins (Fernex, Nagel, & Weber, 2011). To avoid such negative predation effects, strips of more than 10 m width were promoted (only 10 of 82 fallows were narrower than 10 m). Further, because territory density is higher in smaller fallows, high abundances of farmland birds could be reached with strips rather than with parcels of fallows (which is usually the case for rotational fallows in Switzerland), thereby also reducing the conflict with food production. Interactions between edge effects, predation, and management costs are complex and need further study.

The nearer a fallow was established to a wood, the lower was its territory density of studied bird species. Fallows placed 200 m from the nearest wood had six times more territories than areas within 10 m of a wood (Figure 4b). One possible reason for this result is the fact that many locations next to woods are shady and wet. There, the benefit of fallows is impaired due to a reduced botanical diversity as many forbs need sunny locations. A lower botanical diversity has been related to fewer invertebrates (Baines, Hambler, Johnson, Macdonald, & Smith, 1998; Frank, 2000; Pfiffner & Luka, 2000) and so may impair the food resources for most bird species during the breeding season (Benton et al., 2002). Furthermore, some species may avoid fallows in the proximity to wood to minimize the risk of predation from birds nesting in trees (Michel, Jiménez‐Franco, Naef‐Daenzer, & Grüebler, 2016).

Due to sowing or seed pools in the ground, differing soil fertility, vegetation succession and management, fallows show quite a variety of vegetation composition and structural diversity. Our results show that the density of farmland bird territories increases with the proportion of bramble and the occurrence of shrubs in fallows. From experience, we assume that the proportion of bramble in fallows peaks in an optimum with high proportions of bramble being less favorable for most farmland birds. Although we did not find a quadratic effect of bramble, this may arise from the very few samples with high proportions of bramble (only four of 82 fallows had more than 50% bramble, 90% of the fallows showed max. 30% bramble). The effect of shrubs on territory density was quite variable (it had a wide credible interval) and therefore not significant. However, we consider it relevant because it was as strong as the effect of bramble. Its effect may depend on the structure and species composition of the shrubs and would be a topic for further work. It is already known that the Whitethroat favors bramble as breeding habitat, but it seems that some bramble in fallows enhances their attractiveness for several other bird species. Our model estimates an increase of 1.5 territories per ha fallow when the amount of bramble is enhanced from 0% to 10%. Furthermore, predicted territory density increases from 4.3 territories per ha in shrubless fallows to six territories per ha in fallows containing shrubs. Both, bramble and shrubs, are nesting habitat and provide shelter from bad weather and predators, food resources (berries, seed and insects), and song posts or perches. In the Swiss AES directive, bramble and shrubs are permitted in wildflower areas to the amount of 1%. But they are only rarely tolerated by farmers, as these plants can overgrow fallows in a short time, which leads to the withdrawal of AES subsidy payments in Switzerland. We suggest to allow shrubs or bramble on about 10% of the area of fallows and to adapt the Swiss AES in this regard. A similar value of bushes was demanded for pastures in Sweden (Pärt & Söderström, 1999). However, if the conservation focus lies on open farmland species like the Lapwing or Skylark, shrubs must be kept low as these species are known to avoid high structures.

The positive relationship of farmland bird density with the amount of goldenrod is most probably also linked with structural richness. Goldenrod grows taller than other forbs in fallows and therefore serves as a preferred perch or song post for several studied species. Because of its invasive character, it has to be mechanically kept under control in fallows. Ideally, fallows contain enough other, native tall‐growing forbs that remain after winter, for example Wild Teasel *Dipsacus fullonum,* or Dense‐flowered Mullein *Verbascum densiflorum*. Such plants as well as bramble and shrubs increase structural diversity and habitat heterogeneity of fallows, a key factor driving biodiversity (Benton, Vickery, & Wilson, 2003). In practice, tall‐growing forbs should be included in the seed mixes for fallows, as is partially performed in Switzerland. Thorough information, advisory services, appreciation, and financial support are key factors to motivate farmers to change their fallow management and to select sites — for fallows — that optimally benefit biodiversity (Chevillat et al., 2017; Field, Hill, Carroll, & Morris, 2015; Perkins, Maggs, Watson, & Wilson, 2011).

As mentioned in the introduction, fallows are beneficial for biodiversity in general. We expect structural and floristic richness by brambles and shrubs to positively affect a variety of species (i.e., Orthoptera species, Detzel, 1998). A similar result was found for hedges (Graham, Gaulton, Gerard, & Staley, 2018). The occurrence of goldenrod may have negative impacts specifically on invertebrates because they are not adapted to this neophyte. Optimum values of specific measures (i.e., distance to wood, amount of brambles, or bushes) also vary between different species and taxa. Therefore, a diversity of habitats should be promoted at the local scale and management prescriptions should aim at structural richness within and between fallows (Graham et al., 2018; Tscharntke et al., 2011).

## 5 CONCLUSION

Our study reveals that perennial fallows in Switzerland are a suitable option in arable landscapes to benefit different farmland bird species. Yet, location, size, and vegetation composition are important factors. The benefit of fallows can be enhanced through wise site selection and modified management, such that these areas are not established next to woods and are kept structurally diverse, featuring small proportions of brambles, shrubs, and tall‐growing forbs. This can be achieved through a seed‐mix adapted to the region where fallows are planned to be established. Further, cutting is not recommended or only partially and selectively in order to avoid overgrowing by bramble, shrubs, and invasive plants like goldenrod and to keep shrubs low. Advisory services (ideally by local consultants) are necessary and should be available for all farmers within an agri‐environment scheme. Our results are relevant for arable farmland in simple landscapes (less than 20% semi‐natural habitats) in Switzerland and may also in wider continental Europe. The above‐mentioned management measures provide habitat structures which have become very rare in many European countrysides, and which benefit a variety of farmland bird species.

## CONFLICT OF INTEREST

None declared.

## AUTHOR CONTRIBUTIONS

Jérôme Duplain, Michael Lanz, and Simon Birrer gave substantial contribution to the design of the study and the interpretation of the data. Jérôme Duplain and Bernard Lugrin collected the data. Kim Meichtry‐Stier analyzed the data and drafted the article. Jérôme Duplain, Michael Lanz, Bernard Lugrin, and Simon Birrer revised the article, and all co‐authors gave their approval for the final version to publish.

## DATA ACCESSIBILITY

Data on territories numbers and habitat characteristics will be archived on Dryad upon manuscript acceptance.
