# Supplementary material for: The importance of size, location, and vegetation composition of perennial fallows for farmland birds
Source: Ecol Evol. 2018 Aug 24;8(18):9270–81. doi: 10.1002/ece3.4420 (PMC6194211; doi:10.1002/ece3.4420)
Supplement: Supplementary file 1 [file ECE3-8-9270-s001.docx]

**Supporting Information**

**Appendix A1:** Simplified ranking matrices of the compositional analysis for the seven analysed bird species, based on comparing proportions of habitat within pseudoterritories with proportions of available habitat categories in the study site. Breaks of lines represent significant differences between habitat categories.

**Common Whitethroat (n=62)**

habitat fallow hedgerow arable gravel wood others meadow

fallow --------

hedgerow ----------

arable ---------------------- --------

gravel --------------------------------------

wood ----------------------

others -------- ----------------

meadow ---------------- ----------------

**European Stonechat (n = 64)**

habitat fallow hedgerow arable gravel wood meadow others

fallow --------

hedgerow ------------------

arable --------------------------

gravel --------------------------------------

wood ------------------------------

meadow ------------------------------

others ------------------------------

**Melodious Warbler (n=57)**

habitat fallow hedgerow gravel wood arable others meadow

fallow --------

hedgerow ------------------

gravel --------------------------------

wood ----------------------

arable ------------------------------

others ------------------------

meadow ----------------

**Yellowhammer (n=31)**

habitat fallow hedgerow arable wood gravel others meadow

fallow ------------------

hedgerow --------------------------

arable --------------------------------

wood ------------------------------

gravel --------------------------------------

others ------------------------------

meadow ------------------------

**Red-backed Shrike (n=24)**

habitat fallow hedgerow arable gravel wood meadow others

fallow --------

hedgerow ------------------

arable ----------------------------------------

gravel ------------------------------

wood ------------------------------

meadow ------------------------------

others --------

**Corn Bunting (n=22)**

habitat arable fallow hedgerow meadow others wood gravel

arable --------------------------

fallow ------------------------------------------ --------

hedgerow ----------------------------------

meadow ------------------------------------------------

others -------- ------------------------------

wood ------------------------------

gravel -------- ------------------------------

**Skylark (n=80)**

habitat arable hedgerow fallow wood meadow gravel others

arable --------------------------

hedgerow --------------------------

fallow --------------------------

wood --------------

meadow ------------------------------

gravel ------------------------

others ------------------------
